# Sequential and Automatic Image-Sequence Registration of Road Areas Monitored from a Hovering Helicopter

**DOI:** 10.3390/s140916630

**Published:** 2014-09-05

**Authors:** Fatemeh Karimi. Nejadasl, Roderik. Lindenbergh

**Affiliations:** 1 Fugro Intersite B.V., Fugro, Dillenburgsingel 69, 2263 HW Leidschendam, The Netherlands; E-Mail: fkariminejadasl@gmail.com; 2 Department of Geoscience and Remote Sensing, Delft University of Technology, P.O. Box 5048, 2600 GA Delft, The Netherlands

**Keywords:** image sequence stabilization, registration, sequential error removal, homography

## Abstract

In this paper, we propose an automatic and sequential method for the registration of an image sequence of a road area without ignoring scene-induced motion. This method contributes to a larger work, aiming at vehicle tracking. A typical image sequence is recorded from a helicopter hovering above the freeway. The demand for automation is inevitable due to the large number of images and continuous changes in the traffic situation and weather conditions. A framework is designed and implemented for this purpose. The registration errors are removed in a sequential way based on two homography assumptions. First, an approximate registration is obtained, which is efficiently refined in a second step, using a restricted search area. The results of the stabilization framework are demonstrated on an image sequence consisting of 1500 images and show that our method allows a registration between arbitrary images in the sequence with a geometric error of zero in pixel accuracy.

## 1. Introduction

Traffic is a problem in all large cities and is continuously analyzed by both authorities and researchers. Driving behavior is the most influential element in traffic, and its influence on, for example, traffic congestion is poorly understood. One possible approach to obtain insight into driving behavior is to track many vehicles for a long period of time without the drivers' being aware that they are taking part in an experiment [1,2].

For the purpose of studying driving behavior in real traffic situations, a freeway is observed by a camera mounted below a hovering helicopter. The helicopter flies between 300 and 500 m above the freeway and records image sequences for a period of a maximum of a half an hour. The camera used for this purpose has a black and white visual sensor, a frequency of 15 frames per second and a resolution of 1392 × 1040 pixels. An area of 300 to 500 meters was covered on the ground with a spatial resolution of 20 to 50 centimeters. In this paper, a test data set consisting of an image sequence of 1500 images is considered.

The aim of the image sequence analysis is to extract the position of each vehicle in each image where it appears. The instability of the helicopter causes camera motion, which is added to the vehicle motion. The camera motion should, therefore, be separated and eliminated from the vehicle motion. In this way, the result should be a stabilized image sequence.

A large number of images require automatic stabilization. In addition, data are recorded in different traffic situations, surroundings and weather conditions.

The objective of this paper is, therefore, to describe a sequential and automatic framework aiming at the precise registration of a road area for an entire sequence of images.

We design a framework based on two homography assumptions. The first assumption is that the relation between corresponding image coordinates of two consecutive images is expressed by a homography. The second assumption is that also the relation between corresponding image coordinates projected from road points in two arbitrary images is a homography.

The framework is defined as follows:

Coarse road area registration: Coarse road area registration is performed, based on the assumption that the relation between any two consecutive images is a homography.

Precise road area registration: Precise road area registration is performed, based on the assumption that the relation between corresponding points of the current image and the reference image is a homography. Here, only corresponding points on the road area are considered.

In the coarse registration step, an approximate homography of the current image to the reference image on the road area is obtained as the product of the previous homography, the homography between the previous image and the reference image on the road area and the current homography between the current image and the previous image for the whole image area. The current image is transformed based on the resulting approximate homography. As a result, the road area of the current image is registered approximately to the reference image.

In the fine registration step, the road area is extracted once in the reference image. As a result of the coarse registration step, the points on the road area in the current image are very close to their corresponding points in the reference image. The majority of the road area feature points belong to repeated patterns, such as road stripes. The usual feature detection and matching methods, such as SIFT, are not able to correctly match such points. Therefore, we proceed as follows. First, feature points are extracted once from the road area of the reference image. Next, during the fine registration of the current image, these feature points from the reference image are tracked in the transform of the current image. Here, the transform corresponds to the coarse registration as estimated in the coarse registration step. These tracking results are then used to estimate the homography between the reference image and the transformed current image. By going through the image sequence and by multiplying the newly found homography with the homography obtained for the previous current image, sequentially, all images get (fine) registered to the reference image.

After a literature review (Section 2), the problems of current methods are identified in detail (Section 3). The proposed framework is described in Section 4. The results on the test data and discussions are presented in Section 5. The conclusions come in Section 6.

## 2. Literature Review

In an image sequence, the displacement of each pixel across images is camera-induced, scene position-induced and/or independent object motion-induced. If the camera motion is without translation (only rotation and/or zooming) or if the scene is planar, the relation between corresponding points in each pair of images is expressed by a homography without the influence of the scene position [3,4]. For the pixels belonging to moving objects in the scene, the projection of this movement is added to the homography-induced displacement. Computing homographies is faster and less erroneous than the structure from motion (SfM) process [3], because homography parameters are determined by a few corresponding points. The rest of the points in one image are transformed by the calculated homography to the corresponding points in another image. As in the SfM process, outlier removal is an indispensable part of homography parameter estimation.

In Sinha *et al.* [5] and Brown *et al.* [6], the mosaic of the image sequence, which is recorded from a pan-tilt-zoom camera (non-translational motion) is built using a homography model between each pair of images. Bundle adjustment jointly calculates all of the homography parameters from corresponding points to remove the accumulated error due to pairwise homography calculation. Outliers are removed by RANSAC [7]. In Kirchhof *et al.* [8], the scene is assumed to be planar, because of the high altitude. Song *et al.* [9] mosaicked images by ignoring scene-induced motion.

In this setting, the homography relation only holds in the case that the camera motion is without translation and the scene is planar. Scene-induced motion makes the homography model erroneous. The errors become larger whenever the distance to the estimated homography plane increases [10].

In the presence of scene-induced motion, Toth *et al.* [11] orthorectified an image sequence using knowledge on camera motion and a digital surface model (DSM). The camera motion was obtained by a GPS/IMU navigation system, which is good enough for a moving platform with a low frequency camera of 0.2 frames per second (FPS).

The obvious problem of orthorectifying an image sequence using this method is that a DSM of the region should be available and, more importantly, that the image points and object points should be linked manually for the calculation of the camera motion, which is impractical when dealing with a large number of images.

The SfM approach [3] is a possible solution in the case of the unavailability of a DSM and GPS/IMU instruments for measuring camera motion. Camera motion and a textured DSM of a region can be reconstructed by the SfM process using only an image sequence. Corresponding points of all images of the sequence are used to calculate both camera motion and sparse scene coordinates automatically and simultaneously by bundle adjustment, which prevents error propagation during parameter estimation. Then, textured dense scene coordinates are reconstructed by intersecting corresponding points in different images using the previously calculated camera motion. A dense disparity map, an image representing displacements, is created to relate all corresponding points [12]. An alternative method for 3D reconstruction without direct camera motion estimation is the work of Furukawa and Ponse [13] on multiview stereo (MVS) matching and reconstruction. Outlier removal is an inevitable part of the SfM process, because corresponding points must belong to fixed objects only, and moreover, some point matches may be wrong.

An example of an SfM algorithm aiming at image sequence stabilization is described in Liu *et al.* [14]. In this paper, camera poses (camera motion) and sparse 3D coordinates are estimated, followed by camera motion smoothing while preserving image content. A general disadvantage of SfM is that it is often erroneous and computationally expensive. To mitigate this problem, Liu *et al.* [15] used the Kinect, a depth camera, to get additional depth information, which improves the camera motion estimation. A depth camera, however, is not always present and is typically only used in close-range indoor applications.

To handle parallax without applying the SfM procedure, several papers used additional intermediate procedures. Liu *et al.* [16] determined 2D feature trajectories, which were consecutively smoothed to produce a stabilized image sequence of a quality comparable to the results of the 3D method of [14]. Wang *et al.* [17] represented trajectories by Bezier curves. For these methods, long trajectories are required. Liu *et al.* [18] and Bai *et al.* [19] model 3D camera motion by bundling 2D camera motion estimations while maintaining multiple and spatially-variant camera paths. In this way, they are able to model nonlinear parallax-based motion. For this purpose, the image is divided into several grids, and for each grid, 2D camera motion is estimated. Grundmann *et al.* [20] used L1-norm optimization to obtain a smooth camera path while using a 2D homography model.

In our special case, we are not only interested in stabilizing the image sequence, but also in vehicle tracking. The above-mentioned stabilization methods, which handle parallax without using SfM, could be used to stabilize our type of image sequences outside the road area. However, these methods are expected to work less well on the road area, because most available features belong to either repeated patterns (road stripes) or to moving vehicles. As a solution, we therefore propose to design and implement a sequential framework to precisely register an image sequence of a road area in a simple, fast and automatic way without neglecting the parallax effect.

## 3. Problems with Existing Methods

In this section, the specific problems of existing methods for image-sequence stabilization of a road area are described. This will lead to a set of requirements for an automatic stabilization method.

### 3.1. Type of Transformation

The relation between the coordinates of a fixed object in an arbitrary image and the coordinates of the same object in the reference image (for simplicity, assumed to be the first image) is either a homography or a fundamental matrix [4]. The relation between two image coordinates is a homography in either of the two following cases:

- The camera translation, **t**, is zero or **t**/*Z_j,p_* is approaching zero
- The scene is planar or the scene height differences are very small compared to the flight height

A. The translation of a hovering helicopter during 0.1 seconds or less is very small. Let **t** and *Z_j,p_* be the camera translation and the depth of scene point *p* to the *j* – *th* camera, respectively. Then, **t**/*Z_j,p_* is considered zero for very large *Z_j,p_* and small **t** between two consecutive images. The relation between two image coordinates is expressed as follows [3]:

(1) $${\text{x}}_{i,p}=K_{i}RK_{j}^{-1}{\text{x}}_{j,p}+K_{i}\frac{\text{t}}{Z_{j,p}}$$

where **x***_j,p_*, **x***_i,p_*, *K_j_*, *K_i_*, *Z_j,p_* and *R* are, respectively, the homogenous image coordinates of the projected scene point *p* in the *j* – *th* and the *i* – *th* image, the calibration matrices of the *j* – *th* and the *i* – *th* images, the distance of the point *p* from the camera *j* and the rotation matrix between two camera coordinate systems.

For two consecutive images, **t**/*Z_j,p_* is approximately zero. Equation (1) is therefore simplified to the following form:

(2) $${\text{x}}_{i,p}=K_{i}RK_{j}^{-1}{\text{x}}_{j,p}$$

$K_{i}RK_{j}^{-1}$ is a 3 × 3 matrix, which is now a homography.

B. Let **X***_j_*_,_*_p_*, **X̃***_j_*_,_*_p_* be, respectively, the homogeneous and Euclidean coordinates of scene point *p* in the camera coordinate system of the *j* – *th* camera. **n** and *d* are, respectively, the normal and intercept of the scene plane. If the scene is planar (**n**^⊤^**X̃***_j,p_* + *d* = 0), it follows that, **X***_j,p_* = [**X̃***_j,p_* − **n**^⊤^**X̃***_j,p_*/*d*] and ${\tilde{\text{X}}}_{j,p}=Z_{j,p}K_{j}^{-1}{\text{x}}_{j,p}$. In this case, the homography relation is formulated as follows:

(3) $${\text{x}}_{i,p}=K_{i}(R-\frac{\text{t}{\text{n}}^{⊤}}{d})K_{j}^{-1}{\text{x}}_{j,p}$$

In any relation between homogeneous coordinates (Equations (2) and (3)), the equality is up to scale.

**Figure 1.**
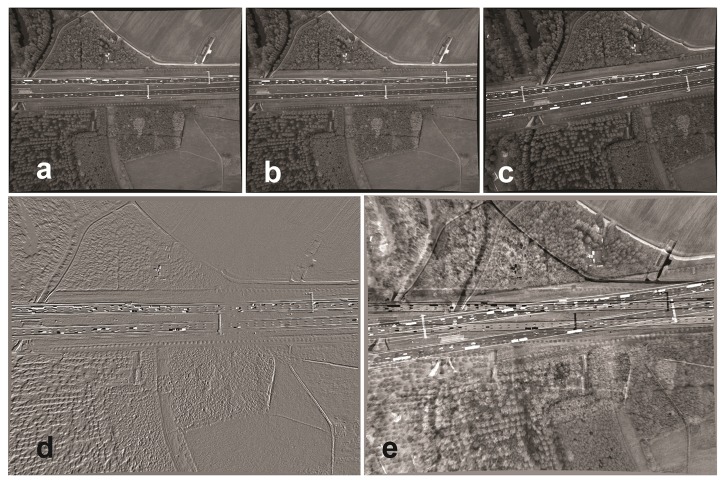
Difference images before registration: (**a**) the first image; (**b**) the second image; (**c**) the 800*th* image; (**d**) the first image subtracted from the second image; (**e**) the first image subtracted from the 800*th* image.

To summarize, the transformation matrix between pixel points is a homography for two consecutive images and a homography or a fundamental matrix for two arbitrary images. Figure 1 shows a very small transformation between two consecutive images (Figure 1d) and a larger one for two arbitrary images (Figure 1e).

Because the road surface in aerial images is locally a plane, the transformation model of the road area is a homography.

### 3.2. Problem in the Parameter Estimation for Each Transformation Type

Only four corresponding points are needed to determine homography parameters. Any three of these points must be non-collinear [3]. Low accuracy of the point localization, however, reduces the accuracy of the estimated homography parameters. More points are therefore required to estimate parameters reliably. Image noise may result in low localization accuracy. Image noise originates from camera noise and image-based problems, such as compression artifacts (all of the images considered in this paper are without compression artifacts, because of using lossless compression.), discretization effects and image blur.

The relation between image coordinate points of two consecutive images (Section 3.1) is parameterized by rotation and calibration matrices (Equation (2)). Consequently, even if the scene points are non-coplanar, the relation of the rotation-based homography holds.

There are two sources of error in rotation-based homography estimation: pixels of moving objects and mismatches. Repeated patterns are one of the main reasons why many point matching algorithms fail. The road area of a freeway severely suffers from this problem, because of road elements, such as stripes and lights. Repeated patterns may also be found in the area outside the road, such as on building blocks or on similar trees. A good initialization, however, reduces the possibility of observing repeated patterns in a limited search area. Outside the road area, there can be few features, such as fields, where finding correspondences is nearly impossible or erroneous. Moving objects not only produce outliers, but also occlude the background.

**Figure 2.**
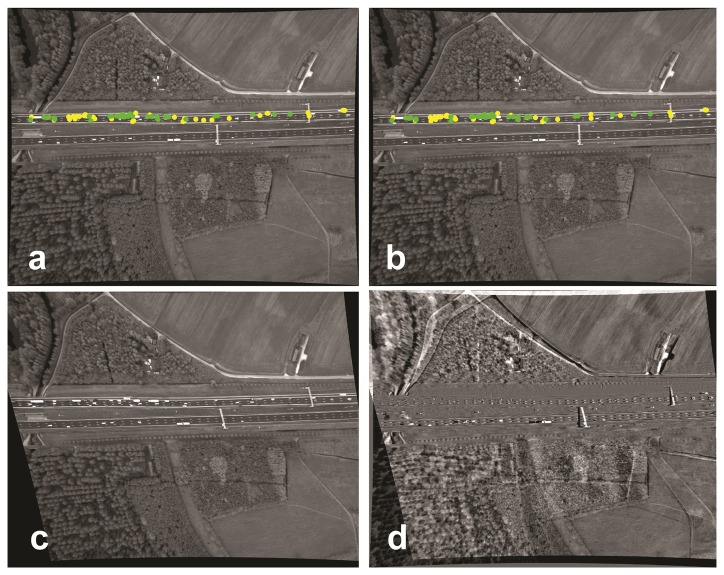
SIFT-based matching results for registration of Image b to Image a. (**a**) The first image of the image sequence; (**b**) the second image. The point colors, yellow and green, are, respectively, the corresponding points matched by SIFT (82) and the remaining corresponding points (41) suitable for the homography matrix calculation; (**c**) The transformed Image b; (**d**) the difference between Image c and Image a.

Even for two consecutive images, using only road area information may be insufficient for road area registration. In an example case, using SIFT [21] for consecutive images from our test data results in a total of 82 candidate corresponding points, but only 41 correspondences remain to determine the homography matrix after applying a RANSAC [7] outlier removal procedure. The registration error by the estimated homography is very large, especially outside the road area (Figure 2).

**Figure 3.**
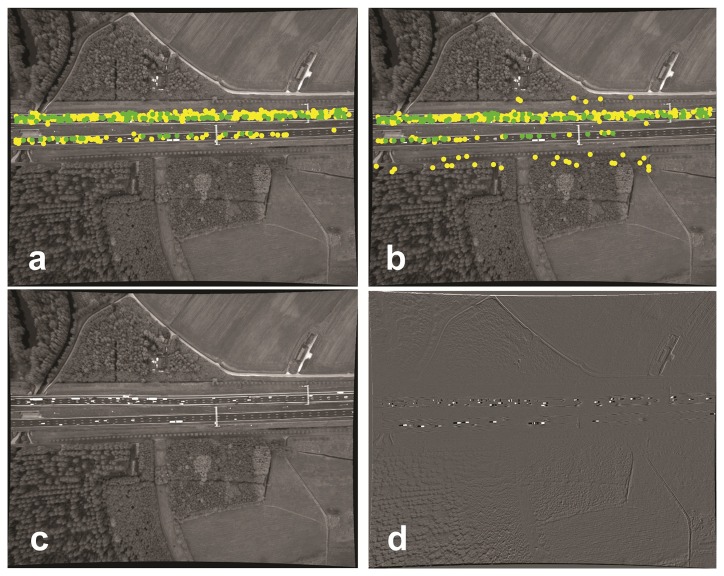
KLT-based matching results for registration of Image b to Image a. (**a**) The first image of the image sequence; (**b**) the second image. The point colors, yellow and green, are, respectively, the corresponding points matched by KLT (229) and the remaining corresponding points (85) suitable for the homography matrix calculation; (**c**) The transformed Image b; (**d**) the difference between Image c and Image a.

Using KLT [22,23] on the consecutive images of our test data results in a total of 299 candidate corresponding points, where 85 of them are detected by RANSAC as inliers to calculate the homography parameters. The registration error by the estimated homography is large outside the road area (Figure 3).

Registration of two arbitrary images has all of the problems of the registration of two consecutive images. In addition, scene points that are not in one plane are another source of outliers if the camera translation is significant compared to the scene depth. The transformation model is, in general, not a homography model.

Moreover, the larger the transformation, the harder the matching is. A large transformation consisting of, e.g., a large scale or rotation may also correspond to viewpoints that differ so much that many matching methods fail. Changing the view occludes or blends parts of the object. Except the accuracy of localization, image noise is also a source of mismatches. Changing light conditions causes additional mismatches. The state-of-the-art local feature extraction and matching methods compete at obtaining correct matches in the case of large transformations, image noise and changing lightening conditions [24].

**Figure 4.**
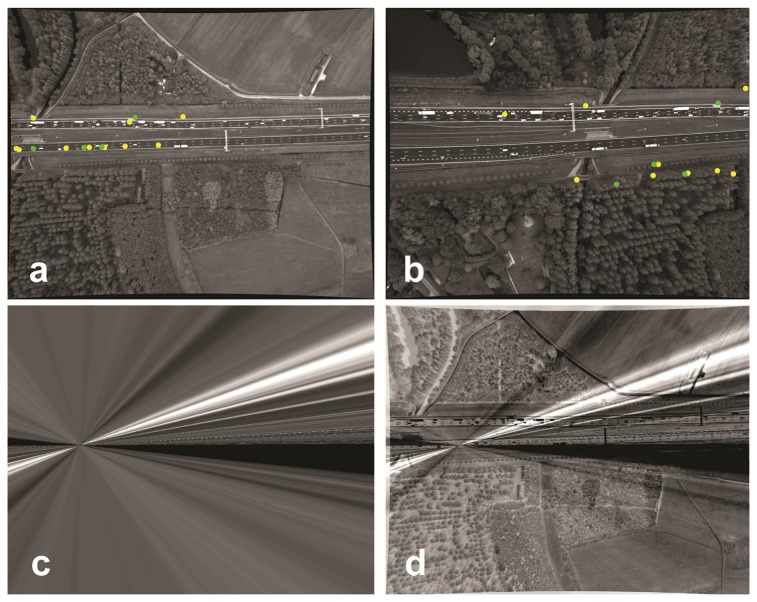
KLT-based matching results for the registration of Image b to Image a. (**a**) The first image of the image sequence; (**b**) the 150*th* image. The point colors, yellow and green, are, respectively, the corresponding points matched by KLT (14) and the remaining corresponding points (4) suitable for the homography matrix calculation; (**c**) The transformed image b; (**d**) the difference between Image c and Image a.

For a test case, we consider two arbitrary images: the left image is the first image of the image sequence, and the right one is the 1500th image from the same image sequence. Only three points are matched between these two images using SIFT; see Figure 4a,b. These points are not even sufficient for the calculation of the homography parameters. Using KLT for point extraction and tracking on the same images for only the road area of the left image results in 14 matched points out of 1500 extracted points. Only four corresponding points out of the 14 matched points are detected as inliers for the homography parameter calculation. By manually checking the remaining corresponding points, it was observed that all 14 points were wrongly matched. The transformed image obtained by applying the estimated homography matrix is, therefore, wrong, as well (Figure 4).

A summary of error sources in image registration of our data is listed in Table 1.

The amount of outliers on the road area is considerably high: moving pixels, repeated patterns and points at different planes, such as lanterns. Restricting the search area to deal with repeated patterns is only effective if the displacement is smaller than the size of the search area. Even for consecutive images, the displacement can exceed the vertical or horizontal distance of the road stripes. A larger search area increases the chance of having repeated patterns, like road stripes. A smaller search area, however, may not contain the points of interest. As a result, to assure the success of this method, the correct size of the search area is vital.

Handling a considerable amount of outliers is the main focus of this paper.

On the test sequence, the existing methods were not satisfactory. That is why we need to design an algorithm to stabilize image sequences of road areas.

## 4. The Image-Sequence Registration Framework

In this section, the detailed framework designed to automatically handle the problems listed in Table 1 is explained. The output of the framework is a stabilized image-sequence of a road area. The image sequences used as an input were recorded from a hovering helicopter over a freeway. The success of the stabilization is guaranteed by a two-step approach, in which, first, the road area of an arbitrary image is transformed to the close vicinity of the reference road area, before fine registration takes place.

### 4.1. Coarse Road Area Registration

The homography $H_{i+1,i}^{c}$ of two consecutive calibrated images (Lines 1 and 5 in Algorithm 1), *I_i_*_+1_ and *I_i_*, is estimated robustly and automatically either by KLT [22,23] or SIFT [21] for finding corresponding features, followed by RANSAC [7] for robust parameter estimation or differential-evolution based (DE-based) registration [25,26]. For DE-based registration, the homography (Equation (2)) is used, requiring the estimation of three parameters only. The results of all methods are reliable, because all of them eliminate the outliers in the entire image area when registering two consecutive images.

The homography $H_{i+1,i}^{c}$ model holds for all image points, excluding the pixels belonging to moving objects:

(4) $${\text{x}}_{i}=H_{i+1,i}^{c}{\text{x}}_{i+1}$$

where **x***_i_* and **x***_i_*_+1_ are homogeneous coordinates in the *i* − *th* and *i* +1 − *th* consecutive images. For each image in a sequence, the homography relation with the previous image is determined (Line 6 in Algorithm 1).

This homography relation between consecutive images holds in particular for the road pixels, ${\text{x}}_{i}^{r}$, ${\text{x}}_{i+1}^{r}$:

(5) $${\text{x}}_{i}^{r}=H_{i+1,i}^{c}{\text{x}}_{i+1}^{r}$$

The road area, ${\text{x}}_{1}^{r}$, is, therefore, extracted just for the first image (Line 2 in Algorithm 1). The first image of the entire sequence is further taken as a reference image. The approximate extraction of the road area in the first image is sufficient for the entire framework, so it can be manually identified. Most road structures present in the helicopter data are straight and can therefore be extracted by, e.g., the method explained in [27].

To improve the numerical stability, the homography matrix is normalized by dividing it by its bottom right element (*h*_33_):

(6) $$h_{ij}=\frac{h_{ij}}{h_{33}}$$

Having identified the road area in the first image, the established homography $H_{2,1}^{c}$ also registers the road area between the first image and the second image:

(7) $${\text{x}}_{1}^{r}=H_{2,1}^{c}{\text{x}}_{2}^{r}$$

The homography $H_{2,1}^{c}$ is equal to the homography $H_{2,1}^{r}$ that relates only the road area of the reference image to the second image ( $H_{2,1}^{r}=H_{2,1}^{c}$). The superscripts *r* and *c* in the homographies denote the homography corresponding to a road plane and the homography between consecutive images, respectively. By transforming the second image ( $I_{2}^{⊤}$) using $H_{2,1}^{c}$, the position of the road area in the first image ( ${\text{x}}_{1}^{r}$) gives, consequently, the position of the road area in the second image.


**Algorithm 1:** Framework of the image-sequence registration of the road area.
 **Input:** Image sequence**1** Calibrate the reference image, *I*_1_**2** Extract the road area in the reference (first) image, 
${\text{x}}_{1}^{r}$**3**
${\tilde{H}}_{1,1}^{r}={ℑ}_{3}$, with ℑ_3_ the 3 × 3 identity matrix.**4 for**
*i*=*first image*+*1*
**to**
*last image*
**do****5**  Calibrate the image *I_i_***6**  Calculate the homography between *I_i_*_−1_ and 
$I_{i}\Rightarrow H_{i,i-1}^{c}$**7**  
${\tilde{H}}_{i,1}^{r}=H_{i-1,1}^{r}H_{i,i-1}^{c}$**8**  
$I_{1}\left({\text{x}}_{1}^{r}\right),I_{i}\left({\tilde{H}}_{i,1}^{r}{\text{x}}_{i}\right)\Rightarrow H_{c}$**9**  
$H_{i,1}^{r}=H_{c}{\tilde{H}}_{i,1}^{r}$**10**  Transform *I_i_* with 
$H_{i,1}^{r}\Rightarrow I_{i}^{\text{T}}$ **Output:** Stabilized image-sequence of the road area.

The relation between the road pixels of the second image and the first image was previously calculated by Equation (7) ( ${\text{x}}_{1}^{r}=H_{2,1}^{r}{\text{x}}_{2}^{r}$). The consecutive homography between the second and the third image is also the homography corresponding to a road plane ( ${\text{x}}_{2}^{r}=H_{3,2}^{c}{\text{x}}_{3}^{r}$). The relation between the first image and the third one on the road area can, in principle, be obtained as the product of two homographies ( ${\text{x}}_{1}^{r}=H_{2,1}^{r}H_{3,2}^{c}{\text{x}}_{3}^{r}$). However, due to error propagation, this relation is in general not accurate enough, and this product is therefore considered to only provide an approximate homography ${\tilde{H}}_{3,1}^{r}$:

(8) $${\tilde{H}}_{3,1}^{r}=H_{2,1}^{r}H_{3,2}^{c}$$

### 4.2. Precise Road Area Registration

In the previous section, it has been described how the road area is approximately registered between the third image and the first image. The third image is transformed by the approximate homography and resulting in $I_{3}^{⊤}$. The homography between the transformed third image and the road area of the first image (*H_c_*) is estimated.

SIFT-RANSAC cannot be used for the purpose of the direct determination of *H_c_*, because of the many moving vehicles, repeated patterns and illumination variations. The same problems may cause a failure of DE-based registration, and therefore, this method should not be used for precise registration of road areas.

*H_c_* is only calculated based on using corresponding points on the road area. All of the possible points are extracted in the first image and then tracked in the transformed third image.

The distinctive road points are tracked either by tracking part of the KLT or by a template matching method in a very limited search area. Each point is tracked separately. Note that, because of the coarse registration, the corresponding points are very close to each other. Large transformations, such as the large rotations, are, therefore, not present, as they were previously removed by the coarse registration step.

The homography *H_c_* is estimated from all corresponding points obtained from template matching. The homography is calculated reliably, because the road area of the third image is very close to the road area of the first image. The search area is, therefore, kept very limited (here 7 × 7 pixels). As a result, repeated patterns are, in general, absent in the search area. The errors caused by pixels belonging to moving objects are very small, in the range of half the search area, and therefore mitigated by the very large number of corresponding points.

The product of this homography and the approximate homography is considered to represent the real homography between the road area in the third and the first image.

(9) $$H_{3,1}^{r}=H_{c}{\tilde{H}}_{3,1}^{r}$$

The third image with the final homography $H_{3,1}^{r}$ is the transformed image $I_{3}^{⊤}$. The position of the road area in the first image is the same for the transformed third image $I_{3}^{⊤}\left({\text{x}}_{1}^{r}\right)$.

The same procedure is used for all images in the sequence to register the images to the first image restricted to the road area.

First, the approximate homography ${\tilde{H}}_{i,1}^{r}$ of the *i* – *th* image is obtained as the product of the homography $H_{i,i-1}^{c}$ between this image and the previous one and the homography $H_{i-1,1}^{r}$ corresponding to the road plane of the previous image to the first image (Line 7 in Algorithm 1):

(10) $${\tilde{H}}_{i,1}^{r}=H_{i-1,1}^{r}H_{i,i-1}^{c}$$

Then, the homography *H_c_* is calculated between the transform of the *i* – *th* image (*I_i_* ( ${\tilde{H}}_{i,1}^{r}{\text{x}}_{i}$) obtained by applying the approximate homography and the road area from the first image (*I*_1_ ( ${\text{x}}_{1}^{r}$) (Line 8 in Algorithm 1).

The final homography $H_{i,1}^{r}$ is therefore the product of the recently calculated homography *H_c_* and the estimated homography (Line 9 in Algorithm 1).

(11) $$H_{i,1}^{r}=H_{c}{\tilde{H}}_{i,1}^{r}$$

The image *I_i_* is transformed by this homography to obtain $I_{i}^{⊤}$ (Line 10 in Algorithm 1), where the road area in the first image is positioned at the same place $\left(I_{i}^{⊤}\left({\text{x}}_{1}^{r}\right)\right)$.

The details of the framework are summarized in Algorithm 1.

If world coordinates are required for the stabilized sequence, the registration of the stabilized sequence to a world coordinate system is obtained using the homography relation between a minimum of four control points on the road surface and the 2D correspondences on one of the images to calculate the homography parameters. This homography model is then used to transform image coordinates to world coordinates for all images. The correspondence between the world points and the image points is obtained manually, but only for one of the images and at least four points.

## 5. Results and Discussion

Our framework is tested on the test data introduced in Section 1. This data set, notably, has the following properties:

- Varying traffic conditions ranging from easily moving, congested, but moving to no movement at all.
- The gantries that were visible in the beginning completely disappeared at the end (Figure 4a,b).
- Non-coplanarity of road points and tree top points in the large transformation; e.g., the transformation between the first and the last image of Figure 4a,b

The helicopter hovers over the freeway and gradually drifts away (Figures 1 and 4a,b).

**Figure 5.**
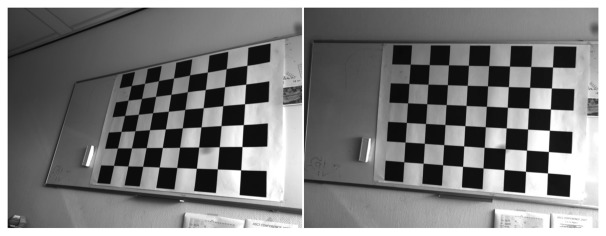
Calibration board from two different camera locations.

In our image sequence, the internal camera parameters remain unchanged. They are, therefore, estimated first. The camera calibration toolbox [28] is used for the camera calibration [29,30]. The grid pattern is specified by a calibration board (Figure 5). The images obtained by changing camera position and orientations are used to estimate the internal camera parameters. Image coordinates are also distorted by lens distortion with radial and tangential components [29,31,32]. The parameters of these two distortions also belong to the internal camera parameters and are estimated in the camera calibration procedure. The image coordinates for homography estimation are used after removal of lens distortion.

After camera calibration, Algorithm 1 was applied to the entire sequence of 1500 images to stabilize the road area. The time required for the registration of the road area of each image to the reference image was approximately three seconds on a normal desktop computer.

### 5.1. Rough Evaluation

To roughly evaluate the stabilization result, three methods were applied: evaluation by movie, by spatio-temporal image assessment and by considering difference images.

#### 5.1.1. Evaluation by Movie

The movie of the image sequence of the road area only shows the stability of the result. This is enough to check if the road area of the image sequence is roughly registered. Gradual error propagation, however, is ignored using the movie evaluation. For example, a video of an image sequence stabilized by the multiplication of consecutive homographies appears very stable, but only when two arbitrary images are compared, differences become visible.

#### 5.1.2. Evaluation by Spatio-Temporal Image

A cross-section in a spatio-temporal image sequence is another way of qualifying the stabilization by visualizing one specific image direction in all images. Each cross-section in the time domain reconstructs an image that is called a spatio-temporal image. Fixed objects appear as vertical lines, while slanted lines correspond to moving objects.

**Figure 6.**
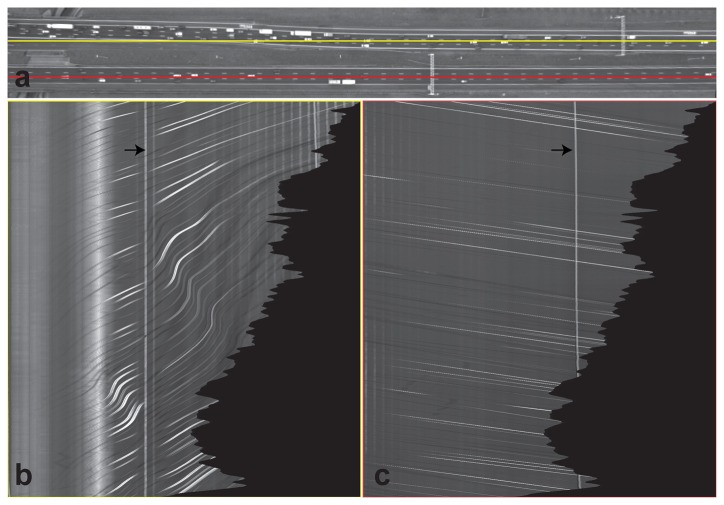
A spatio-temporal image. (**a**) One image from the image sequence. The x-axis is chosen, such that it is parallel to the road line direction of the lowest road line presented in red and top road in yellow. (**b,c**) Spatio-temporal images showing the pixels on the red and yellow lines at the location indicated in the top image as a function of time. The x-and y-axis of this image are, respectively, the image line direction and the temporal direction. The time is increasing downwards. The gantries' locations are indicated in (a) and (b) by arrows.

Spatio-temporal images of a line from both sides of the road area are selected (red and yellow lines in Figure 6). The vertical lines in the spatio-temporal images represent fixed objects, while the slanted lines correspond to vehicles. The black area is the area without any information. The line corresponding to the gantry, indicated by the arrow, is slightly slanted, which is caused by parallax movement. The points on the top of the gantry are located in a different plane than the road points. This line appears as the brightest line in the red spatio-temporal image.

#### 5.1.3. Evaluation by Difference Images

**Figure 7.**
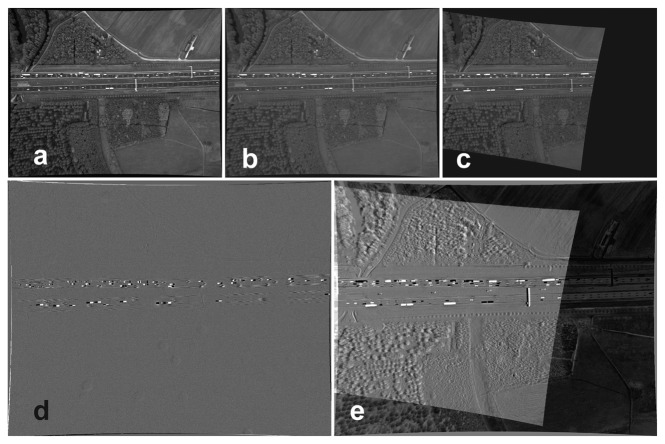
Difference images after registration: (**a**) the reference image (first image); (**b**) the transformed second image; (**c**) the transformed 800*th* image; (**d**) the first image subtracted from the transformed second image; (**e**) the first image subtracted from the transformed 800*th* image.

To form a difference image, the corresponding gray level of the reference image is subtracted from the transformed candidate image.

(12) $$dI_{i,1}(x,y)=I_{i}^{⊤}(x,y)-I_{1}(x,y)$$

where *dI_i,_*_1_, $I_{i}^{⊤}$ and *I*_1_ are, respectively, the difference image, the transformed candidate image and the reference image. *dI_i,_*_1_(*x*, *y*) highlights the differences between two images. Some difference images are visualized in Figure 1 before registration and in Figure 7 after registration. The images are all subtracted from the reference image. As can be seen in Figure 7, the entire image area of the second image (consecutive image) is registered to the first image. The moving vehicles are highlighted by darker or brighter regions. For Image 800, only the road areas are registered. Pixels that do not belong to the road plane are misplaced. These pixels are in particular located on tree canopies and gantries. The differences in gray values of some road lines and road stripes are caused by their high reflectivity, which changes their gray levels. Evaluation by difference images is particularly used for the evaluation of consecutive images where illumination variation is negligible. It is still a good method for visual evaluation of arbitrary images with lower illumination variation.

### 5.2. Precise Evaluation

After being convinced that the image sequence is roughly stabilized, the stabilized image-sequence is quantitatively assessed and checked for how precisely the road area was stabilized. Defining the precision of the stabilization is, however, difficult. The main difficulties are listed below:

- Because the correct homography parameter values are unknown, the estimated homography parameter values cannot be evaluated against the ground truth.
- The goodness of image registration can be measured using a similarity metric, such as the root mean square of the difference image (RMS) or the peak signal-to-noise ratio of the difference image (PSNR) [33]. The difference image is, however, sensitive to moving objects and illumination variations and, therefore, cannot be used for assessing the registration between arbitrary images from the registered image sequence. Only consecutive images are assessed by this method. As an example, the RMS of the difference image between the first and second image before and after registration is 7.367 and 3.450 units of image intensity, respectively. A similar result of 6.271 *versus* 3.305 units is obtained for the difference between the second and the third image. The RMS error is therefore reduced after image registration.
- The geometric error, the geometrical differences at corresponding pixels, is an alternative way to quantitatively evaluate stabilization results. The difference in the coordinates of corresponding pixels is a geometric error caused by the error in the estimated homography parameter values. After stabilization, corresponding pixels should have the same coordinates. The corresponding pixels used for identifying a geometric error should be uniformly distributed over the common area. Usually, geometric errors are larger in the image corners than in the image center. Corresponding pixels can be obtained manually or automatically. The automatic identification of corresponding pixels might be unreliable, because of moving objects and mismatches. These problems affect the evaluation and lead to a wrong evaluation results. It is, however, impractical to use a manual geometric-error test for an entire sequence.

To make the evaluation tractable, two images are selected randomly from the image sequence. For each pair of images, the registration is first evaluated by flipping the visualized images. Then, the geometric-error test is applied on a few randomly selected pixels and also on pixels that seem to move based on visual inspection.

**Figure 8.**
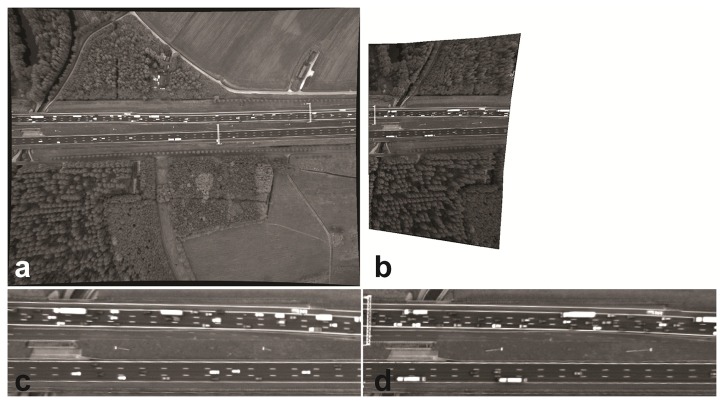
The registration result of the framework: (**a**) the reference image (first image) and (**b**) the transformed 1500*th* image obtained with the registration procedure described in this paper. The non-overlapping area is removed from the 1500*th* image. The road area is visualized in (**c**) and (**d**) in the same order. The geometric errors for the corresponding points on the road area are zero in pixel accuracy.

Here, we selected the first and the transformed last image of the sequence (Figure 8). Figure 8 shows the large geometric error for canopies, gantries and lanterns. This is due to the difference in viewpoint from the platform. As an example, consider the gantry that is visible on the left part of the road in the transformed 1500*th* image: this gantry is visible on the road area common to the 1500*th* image (Figure 8d), but not in the corresponding road area of the first image itself (Figure 8c). The geometric error is zero up to pixel accuracy for all of the points on the road surface. Because of having the same image coordinates for pixels on the road area, the coordinates were not displayed. The difference image of the road area was uninformative due to illumination variation.

**Figure 9.**
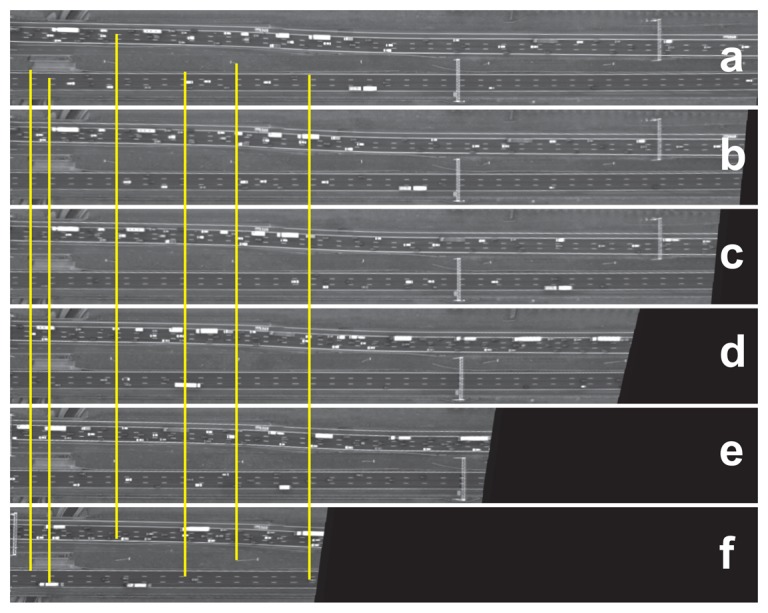
The registration result of the framework: (**a**) Image 1; (**b**) 15; (**c**) 59; (**d**) 661; (**e**) 991; (**f**) 1500. The yellow lines connect corresponding points.

**Figure 10.**
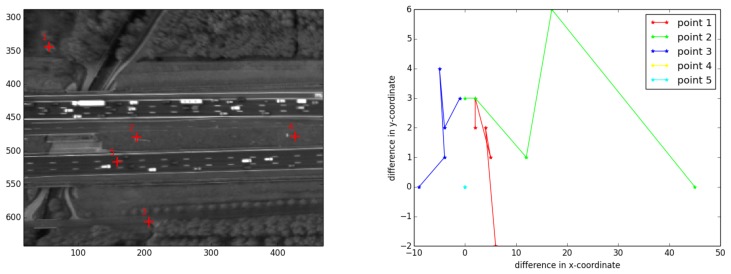
The red crosses indicate five manually-selected points (**left**). Points 1–3 are located outside the road plane, while Points 4 and 5 are located on the road plane. These points are matched between Images b–f and reference Image a, all shown in Figure 9. The difference in x-and y-coordinates in pixels between the matched points and the coordinates of the corresponding points in the first image are visualized in (**right**).

Many other images were selected and compared to the first image. For all of these images, the geometric error of the road points was zero. For the sake of visualization, a few random frames of the road area are vertically displayed in Figure 9, and corresponding points are connected by yellow lines. To qualitatively present the same result, we selected five points manually (Figure 10a) and assessed their correspondences in the images shown in Figure 9. The first three points were from outside the road plane, while the last two do belong to the road. The differences of their x-and y-coordinates to the reference image are computed and visualized in Figure 10b. As can be seen, the first three points have larger geometric errors, because they are not located on the road plane, while the last two points, located on the road plane, have zero error.

It is concluded that the image sequence is precisely stabilized for all of the pixels on the road area. A summary of the above-mentioned methods is given in Table 2.

## 6. Conclusions

The framework described in this paper stabilized an image sequence of a road area in an automatic and sequential way. The stabilization result is reliable, because the very closely approximated homography guarantees the precise registration of the road area. Any arbitrary image is registered to the reference image within a geometric error of zero pixels.

KLT-RANSAC is chosen to register the consecutive images because of its speed. Regardless of speed, the consecutive images are registered by SIFT-RANSAC or DE-based registration using rotation-based homography.

An errorless solution of the stabilization procedure makes it unnecessary to jointly estimate all homographies by the expensive procedure of bundle adjustment. The new procedure described in this paper therefore stabilizes the images in a sequential way.

The approach presented in this paper can also be applied to image sequences obtained by other hovering platforms, such as unmanned aerial vehicles (UAV) and autonomous underwater vehicles (AUV). Cameras installed on gantries for traffic monitoring could suffer from vibration, due to wind, which disturbs the tracking procedure. The framework presented here can also be applied directly for these cases. On road crossings, the framework should run separately for each road area. The procedure for each road area is independent and, therefore, can be computed in parallel. Our sequential framework cannot be directly used for the case of a moving platform. After a few frames, the reference frame should be updated. This procedure might not be reliable for such a scenario, because the homography condition is not valid.

## Figures and Tables

**Table 1.** Error sources in the image registration of our data.

Errors Sources	Consecutive Image Pair	Arbitrary Image Pair
Pixels on moving objects	Yes	Yes
Pixels corresponding to non-coplanar scene points	No	Yes
Changing light conditions	No	Yes
Image noise: camera noise, discretization effects and image blur	Yes	Yes
Repeated patterns	Yes	Yes
Scene with sparse structure	Yes	Yes
Large transformation: viewpoint differences, large rotation or scale differences	No	Yes
Scene occlusion due to either large transformations or moving objects	Yes	Yes

**Table 2.** Summary of the methods used to evaluate image-sequence registration.

Methods	Result
**Rough Evaluation**	
movie	image sequence appears to be stable, but the test is not conclusive, because small error growth cannot be observed
spatio-temporal image	moving objects appear as slanted lines and fixed objects as vertical lines
difference image	darker and lighter areas represent non-registered areas; good for visually comparing consecutive images
**Precise Evaluation**	
errors in estimated parameters	not possible to assess, because there is no ground truth available
radiometric errors	lower errors considered as a better match; suited for consecutive images and images with negligible brightness variation; we estimated homography parameters between consecutive images by minimizing this error (Section 4.1, our paper [26])
geometric errors	flipping images and randomly checking coordinates; this method gives the final result
